# Correlation between CT chest severity score (CT-SS) and ABO blood group system in Egyptian patients with COVID-19

**DOI:** 10.1186/s43055-021-00571-5

**Published:** 2021-08-04

**Authors:** Mohamed G. Mansour, Ahmed S. Abdelrahman, Emad H. Abdeldayem

**Affiliations:** Radiology Department, Faculty of Medicine, Ain Shams University, Cairo, Egypt

**Keywords:** CT severity score, COVID-19 pneumonia, ABO blood group

## Abstract

**Background:**

The 2019 coronavirus disease (COVID-19) has become a global health crisis. CT chest is considered as an important investigation for early diagnosis as well as assessment of severity of COVID-19 pneumonia. Several articles reported that there is a correlation between ABO blood group system and susceptibility as well as prognosis of the disease. In our study we correlated the CT severity score (CT-SS) and the ABO blood group in patients with COVID-19 infection. This study involved 547 symptomatic patients with pathologically proven COVID-19 infection (positive PCR); non contrast CT chest was done for all cases and CT severity score (CT-SS) was calculated followed by its correlation with the patients’ ABO blood group. Aim of the work was to evaluate the relation between CT-SS and the ABO blood groups in Egyptian patients with COVID-19 infection.

**Results:**

The mean CT-SS in patients with blood group A patients (*n* = 153; 28%) was 13.7 (moderate severity), while in patients with blood group O (*n* = 227; 41.5%) the mean CT-SS was 6.7 (mild severity). In blood group B patients (*n* = 139; 25.4%) the mean CT-SS was 9.1 (mild to moderate severity) and in blood group AB patients (*n* = 28; 5.1%) the mean CT-SS was 9.7 (mild to moderate severity).

**Conclusion:**

COVID-19 patients with blood group A are more prone to aggressive CT findings (higher CT-SS) and consequently may be susceptible to increased risk of mortality compared to the patients with other blood groups; however, patients with blood group O are suggested to have the least CT-SS and appear to be relatively protected.

## Background

COVID-19 pneumonia is a rapidly spreading acute respiratory syndrome [1]. The most common symptoms are dry cough, fever, anosmia, ageusia and/or fatigue yet, other symptoms such as sore throat, arthralgia, myalgia, and diarrhea may occur. About 5–15% of patients develop a more serious illness that may rapidly progress to respiratory failure with a 2–3% mortality rate [2–4].

At present, the diagnosis of COVID-19 depends on real time reverse transcription polymerase chain reaction (RT-PCR); yet it has some limitations that may cause false negative results. High resolution CT examination is a non-invasive, simple and rapid procedure that can be used as a screening tool for suspected patients as well as provide an objective assessment about the extension of the lung disease and consequently assess the disease burden. CT manifestations are similar to those seen in viral pneumonias, with multifocal ground-glass opacities and consolidation in a peripheral sub-pleural distribution being the most common findings. CT severity score (CT-SS) of the chest is considered as a semiquantitative indicator of lung affection in COVID-19 to assess the disease burden [5–8].

The human ABO blood group system includes four blood types, namely, A, B, AB, and O. Many studies have found that the ABO blood group system plays an important role in various infectious and non-infectious human diseases. Differences in the blood group antigens located on the surface of human red blood cells can increase or decrease host susceptibility to many infections as it can facilitate intracellular uptake, signal transmission, or adhesion and modify the immune response to infection. Because COVID-19 is a new virus, it is unclear whether the ABO blood groups affect severity of COVID-19 disease or not [9–12].

This study aimed to assess the relation between CT-SS and the ABO blood groups in Egyptian patients with COVID-19 infection.

## Methods

All our patients were referred from the chest clinics with positive RT-PCR for COVID-19 infection. CT chest without contrast using a Siemens 16 slice (Siemens Healthineers, Erlangen, Germany), was done for our 547 symptomatic patients (312 males; 57% and 235 females; 43%), to detect if there was pulmonary involvement by COVID-19 infection and the CT severity scoring was calculated, throughout a period extending from May 2020 to December 2020. A radiologist with 20 years of experience in CT chest imaging performed the CT image analysis. All individuals were subjected to clinical and laboratory assessments. Clinical features involved age, gender and disease symptoms such as fever, dry cough, dyspnea, anosmia, ageusia, fatigue and diarrhea. Laboratory findings included ABO blood group test, CBC, CRP, ESR, LDH, D dimer, S. ferritin. An informed consent was obtained from all participants in this study. Confidentiality of all patients’ data was guaranteed. All information was obtained and analyzed with the Excel program.

### Inclusion criteria

- Symptomatic patients with positive PCR examination.
- CT examination was done from the 5th to 13th days after onset of symptoms to detect the most diagnostic CT findings during the progressive and peak stages.

### Exclusion criteria

- Patients with lung masses, history of interstitial lung disease, previous chest surgery, TB or pleural disease.
- Patients with co-morbidities including those with hepatic and renal impairment, heart failure, uncontrolled diabetes and patients with malignancy.

Each lobe could be awarded a CT score from 0 to 5, depending on the percentage of t involved lobe: score 0—0% involvement; score 1—less than 5% involvement; score 2—5 to 25% involvement; score 3—26 to 49% involvement; score 4—50 to 75% involvement; score 5—greater than 75% involvement. The overall CT score was the sum of the points from each lobe and ranges from 0 to 25 points with sensitivity and specificity of 80.0% and 82.8%, respectively [13].

### Statistical analysis

The Statistical Package for the Social Sciences (version 24; IBM Corp., Armonk, NY, USA) was utilized in the data manipulation and significance testing. The patients were classified into three groups according to their CT-SS: those with CT-SS of less than 9 were considered mild, whereas those with CT-SS of 9–15 were considered moderate and those with CT-SS of more than 15 were considered severe. The Chi-square correlation analysis was conducted, and *p* values of < 0.1 were used to denote statistical significance.

## Results

During the study period, 547 symptomatic patients with positive PCR test for COVID-19 (312 males; 57% and 235 females; 43%) underwent lab investigations including blood group test, CBC, CRP, ESR, LDH, D dimer, S. ferritin. High resolution CT chest without contrast was done for all of them and the CT-SS was calculated. Of these, the mean CT-SS in patients with blood group A patients (*n* = 153; 28%) was 13.7 (moderate severity), while in blood group AB patients (*n* = 28; 5.1%) the mean CT-SS was 9.7 (mild to moderate severity) and in blood group B patients (*n* = 139; 25.4%) the mean CT-SS was 9.1 (mild to moderate severity). In patients with blood group O (*n* = 227; 41.5%) the mean CT-SS was 6.7 (mild severity). The mean CT-SS for all patients was 9.9.

### Non-contrast CT chest findings

The most common CT chest findings were the presence of ground glass opacification and consolidation. Less commonly; crazy-paving, halo, and reversed halo signs, subpleural sparing, interlobular septal thickening, bronchial thickening, pleural thickening and rarely minimal pleural effusion.

The demographic features regarding patients’ age and gender in relation to their blood groups are seen in Table 1.

**Table 1:** Patients’ age and sex in relation to their blood groups

	Blood group A	Blood group B	Blood group AB	Blood group O
Mean age	52.7	43.3	47.5	51.6
*Sex*				
Males	87	79	16	130
Females	66	60	12	97

The main symptoms of patients as well as the number of patients of each blood type and the significance of symptoms severity were clarified in Table 2.

**Table 2:** The common presenting symptoms, number of cases in each blood group, and significance of severity

	Blood group A	Blood group B	Blood group AB	Blood group O	*p* value
Dry cough	132	72	22	150	0.075*
Fever	143	122	24	139	0.473
Dyspnea	99	67	7	42	0.062*
Chest pain	126	86	17	68	0.091*
Anosmia	83	85	15	165	0.124
Fatigue	149	121	19	194	0.352
Abdominal pain and diarrhea	44	32	5	74	0.275

*p value < 0.1: significant

**Fig. 1:**
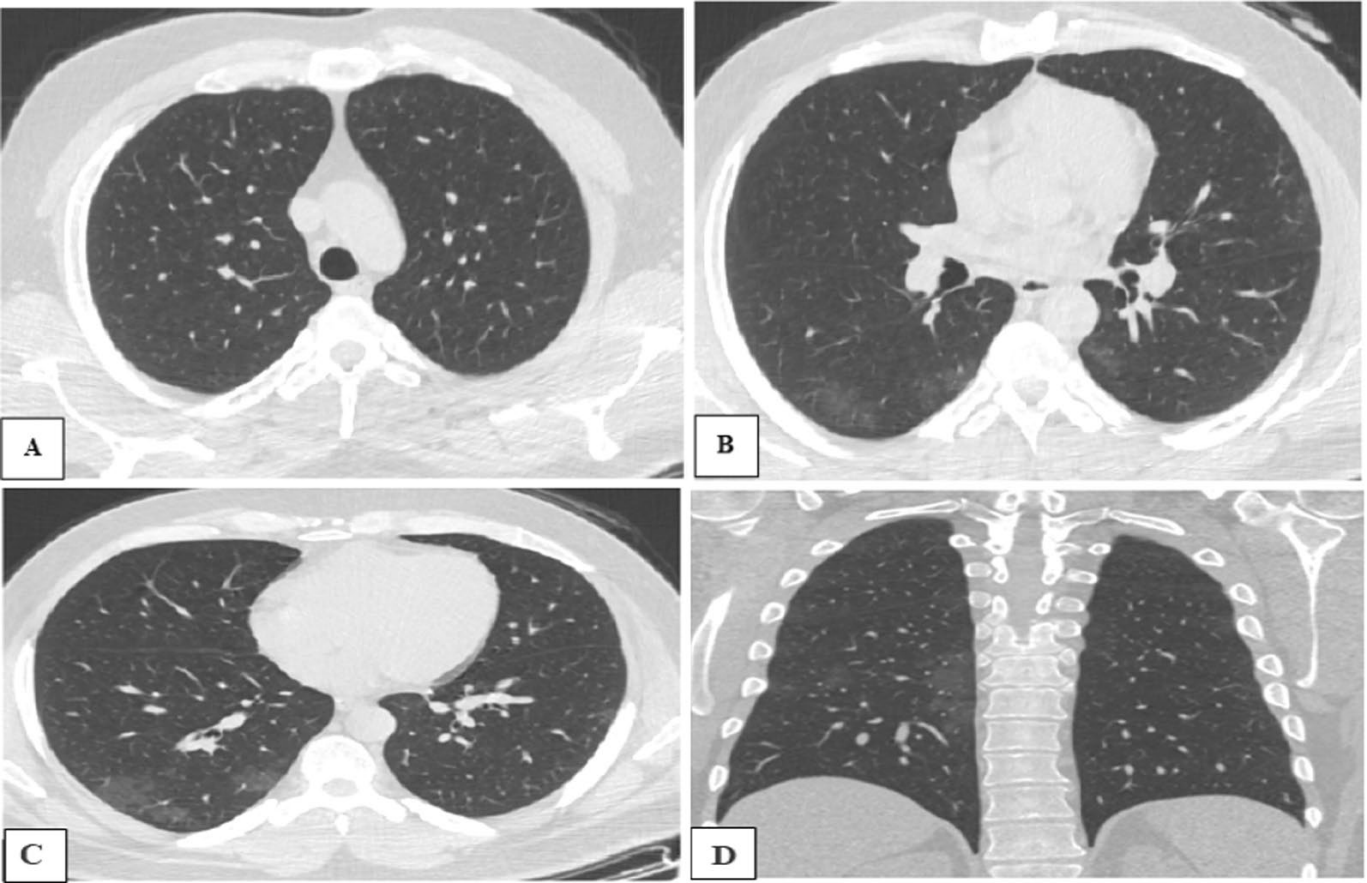
51-years old male patient, with blood group O, having COVID-19 pneumonia, and referred for CT chest assessment. **A**–**C** Axial and **D** coronal CT chest images shows bilateral sub-pleural variable-sized ground-glass opacities. Calculated CT severity score was 4 and such case was considered mild severity

**Fig. 2:**
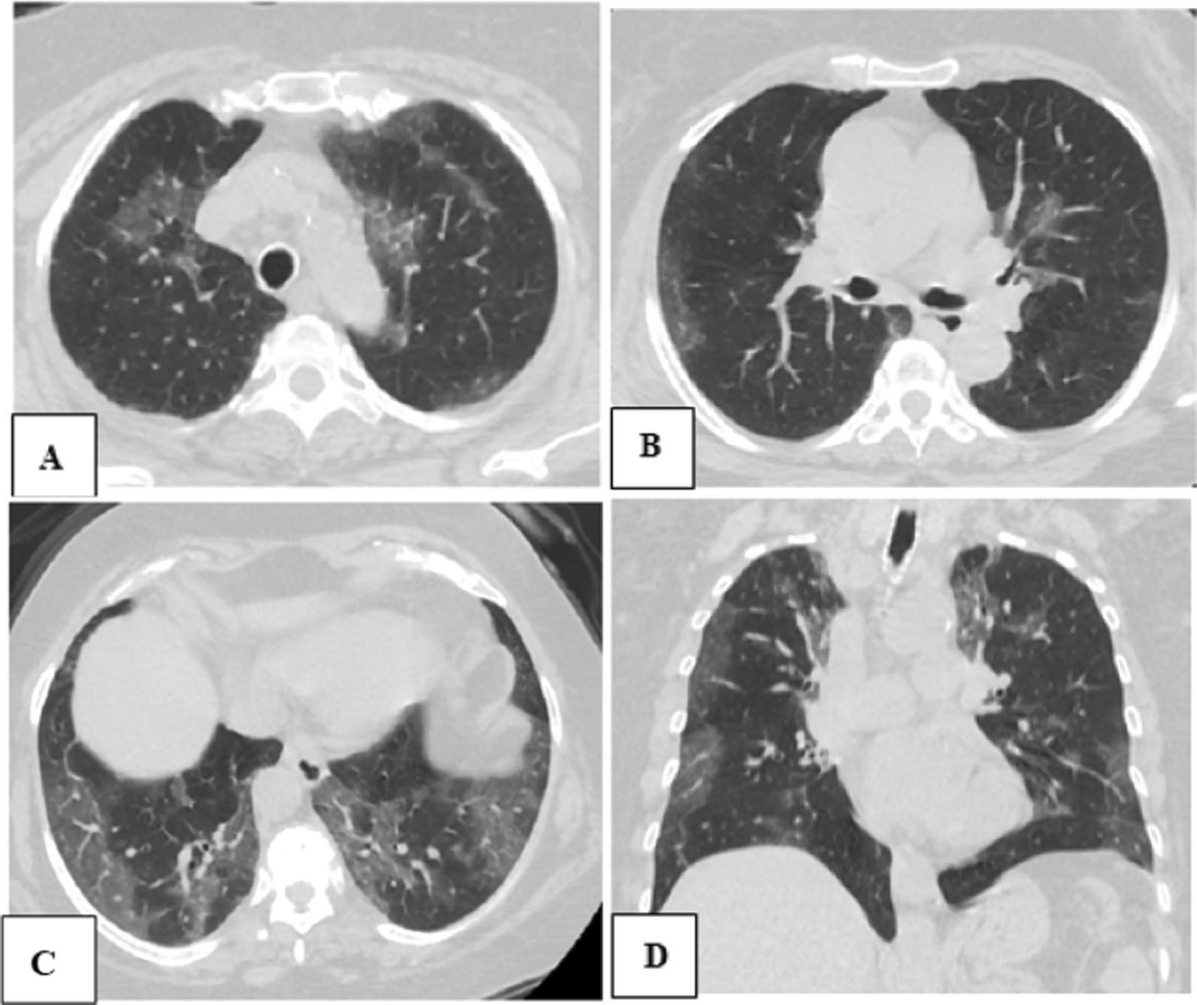
68-years old female patient, with blood group AB, having COVID-19 pneumonia, and referred for CT chest assessment. **A**–**C** Axial and **D** coronal CT chest images shows bilateral sub-pleural variable-sized ground-glass opacities with interlobar septal thickening. Calculated CT severity score was 15 and such case was considered moderate severity

**Fig. 3:**
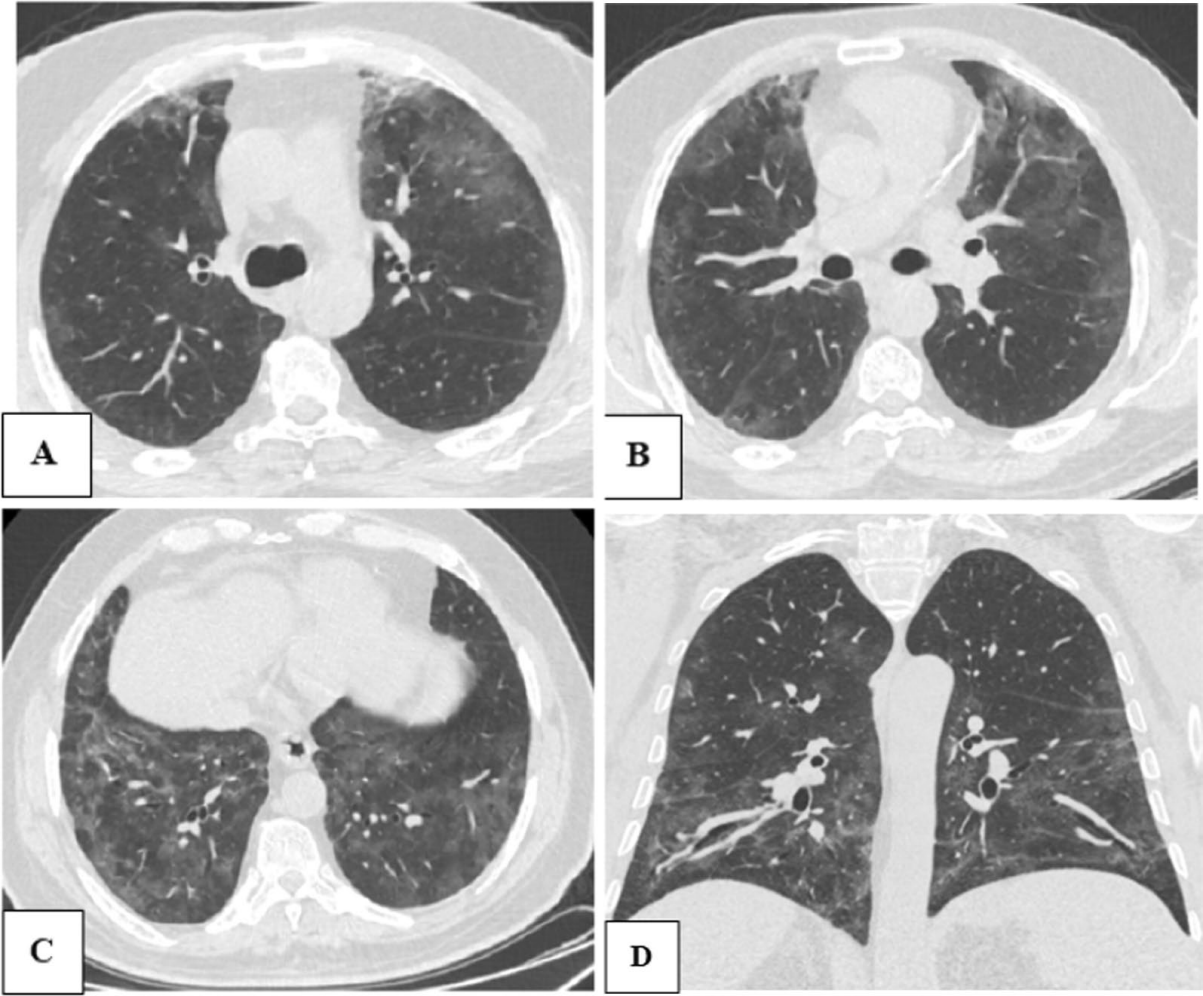
64-years old male patient, with blood group A, having COVID-19 pneumonia, and referred for CT chest assessment. **A**–**C** Axial and **D** coronal CT chest images shows bilateral sub-pleural variable-sized ground-glass opacities with interlobar septal thickening and crazy paving appearance. Calculated CT severity score was 16 and such case was considered high severity

The blood type A and O were determined to have the highest and least mean CT-SS with *p* value of 0.017 and 0.049 respectively suggesting strong correlation between these two blood groups and severity of COVID-19 pneumonia (Table 3; Figs. 1, 2, 3).

**Table 3:** Correlation between the mean CT-SS and the ABO blood groups

	Mean CT-SS	*p* value
Blood group A	13.7	0.017*
Blood group B	9.1	0.235
Blood group AB	9.7	0.194
Blood group O	6.7	0.049*

*p value < 0.1: significant

## Discussion

In this study there was an obvious association noted between ABO blood type and the severity of COVID-19 pneumonia known by calculation of the CT-SS, as we found that there is a strong correlation between the patients with blood group A and the more severe pneumonic process in their non-contrast high resolution CT chest with relatively higher severity score, compared to the other patients with other blood group.

On the other hand, patients with blood group O were found to be relatively protected with less severity of CT findings and less CT-SS which is considered as a mirror to the overall severity of the disease. Most of the other studies correlated the ABO blood group with the susceptibility to the COVID-19 infection rather than the severity of the disease.

However, few articles studied the association between the blood type and the severity of the clinical symptoms of the disease and not with the CT severity scoring system. We found that the patients with blood group A were prone to higher CT severity score and consequently increased risk of morbidity and mortality compared to other blood groups.

The CT-SS was created as a semi-quantitative method to assess COVID-19 burden on the initial CT scan obtained at admission and provide an approach to identify patients in need of hospital admission [14]. The (CT-SS) is a modification of a method used during the SARS epidemic of 2005 [15]. Both lungs were divided into five lobes, and each lobe was assessed individually. The abnormalities that were considered significant for the disease included the following: ground-glass opacity, consolidation, reticulation, crazy-paving pattern, nodule, interlobular septal thickening, linear opacities, subpleural curvilinear line and/or bronchial wall thickening.

Kibler et al., studied the risk and severity of COVID-19 and ABO blood group in Transcatheter aortic valve patients and found that, in these patients the subgroup with the A blood type was especially prone to develop the disease and showed unfavorable outcomes [16].

Zhao et al. in the Wuhan experience evaluated the association between blood type and susceptibility to COVID-19 infection and found that blood group A was associated with a higher risk for acquiring COVID-19 compared with non-A blood groups, whereas blood group O was associated with a lower risk for the infection compared with non-O blood groups [17].

Latz et al., studied the relation between blood groups and the severity of the COVID-19 disease that defined as patient intubation or death and was found that, blood type is not associated with risk of progression to severe disease requiring intubation or causing death, nor is it associated with higher peak levels of inflammatory markers [18].

Liu et al., also evaluated the impact of ABO blood group on COVID-19 infection risk and mortality and found that blood groups A and B may be risk factors for COVID-19 with possible unfavorable outcomes in the group A patients, whereas the blood group O appears to be protective [19].

In our study, we used the CT-SS as a semiquantitative measure to assess the severity of the COVID-19 disease and its impact on the lungs in Egyptian patients and the mean CT-SS was 9.9.

In 2020, Hafez studied the mean CT-SS and its correlation with chest manifestations in Egyptian patients with COVID-2019 pneumonia and stated that the assessment of the CT severity score of COVID-19 is essential for the extent of pneumonia to allow early diagnosis and accurate treatment and found that the mean CT-SS was 11.2 [20].

In February 2020, Yang et al., also used the CT-SS as a tool to assess the severity of COVID-19 pneumonia and found that CT-SS could be potentially used to expedite triage of patients in need of hospital admission [21].

We think that the CT severity scoring may help the clinician in their management of COVID-19 infection, hand by hand with the clinical severity scoring dealing with different aspect of clinical data of the patients including heart rate, blood pressure, temperature, performance, age, sex respiratory rate, O2 saturation, and alertness.

## Conclusion

The blood group A is considered to be more prone to a severer form of COVID-19 pneumonia denoted by higher CT-SS and consequently may be susceptible to increased risk of mortality compared to the other blood groups; however, patients with blood group O are suggested to have a relatively less CT-SS with likely less morbidity and mortality.

## Study limitations

Contribution from multi-center and sharing experience with other countries will be of additive value. The AB blood group is considered the least common blood group worldwide. Among the Egyptian population, the percentage of AB blood group was found to be about 4–10% in the most recent studies. In our chosen sample the percent of AB blood group was 5.1% (28 patients) which was matching with the average percentage among the Egyptian population.

## Data Availability

All the datasets used and analyzed in this study are available with the corresponding author on reasonable request.

## Abbreviations

COVID: Coronavirus disease
CT: Computed tomography
CT-SS: Computed tomography severity score
SARS: Severe acute respiratory syndrome
RT-PCR: Reverse transcription polymerase chain reaction
